# Supplementary material for: Expanded Electroluminescence in High Load CdS Nanocrystals PVK-Based LEDs
Source: Nanomaterials (Basel). 2019 Aug 28;9(9):1212. doi: 10.3390/nano9091212 (PMC6780533; doi:10.3390/nano9091212)
Supplement: Supplementary file 1 [file nanomaterials-09-01212-s001.pdf]

Supporting Information

Expanded Electroluminescence in High Load CdS Nanocrystals PVK-Based LEDs

Fernando Rodríguez-Mas *, Juan Carlos Ferrer, José Luis Alonso and Susana Fernández de Ávila *

Communications Engineering Department, Universidad Miguel Hernández, 03202 Elche, Spain
* Correspondence: s.fdezavila@umh.es (S.F.d.Á.); fernando.rodriguezm@umh.es (F.R.-M.); Tel.: +34-966-658-719 (S.F.d.Á.&F.R.-M.)

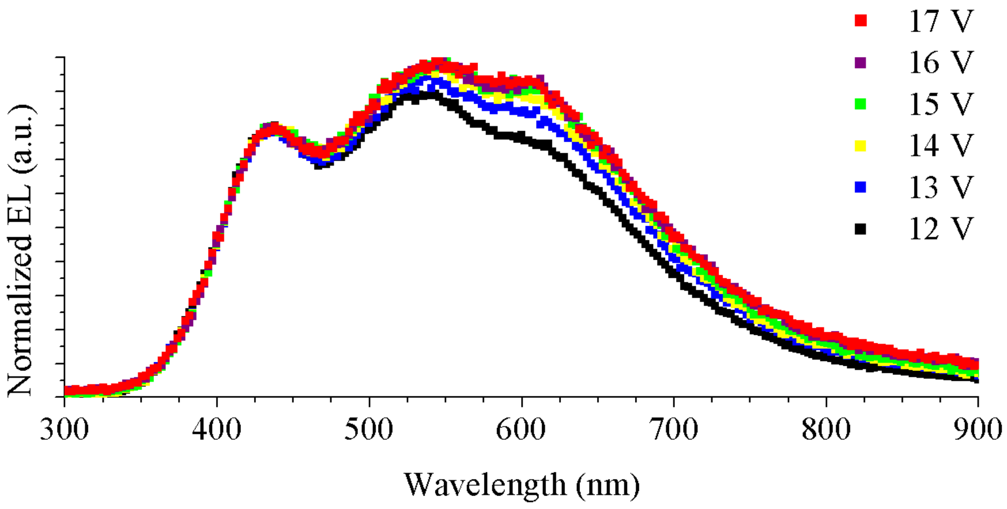

Figure S1. Electroluminescent behavior of the hybrid LED with PVK:CdS NCs [1:0.5] as a function of voltage. Voltage increasing from 12 to 17 V.

All spectra have been normalized to the maximum of PVK emission.

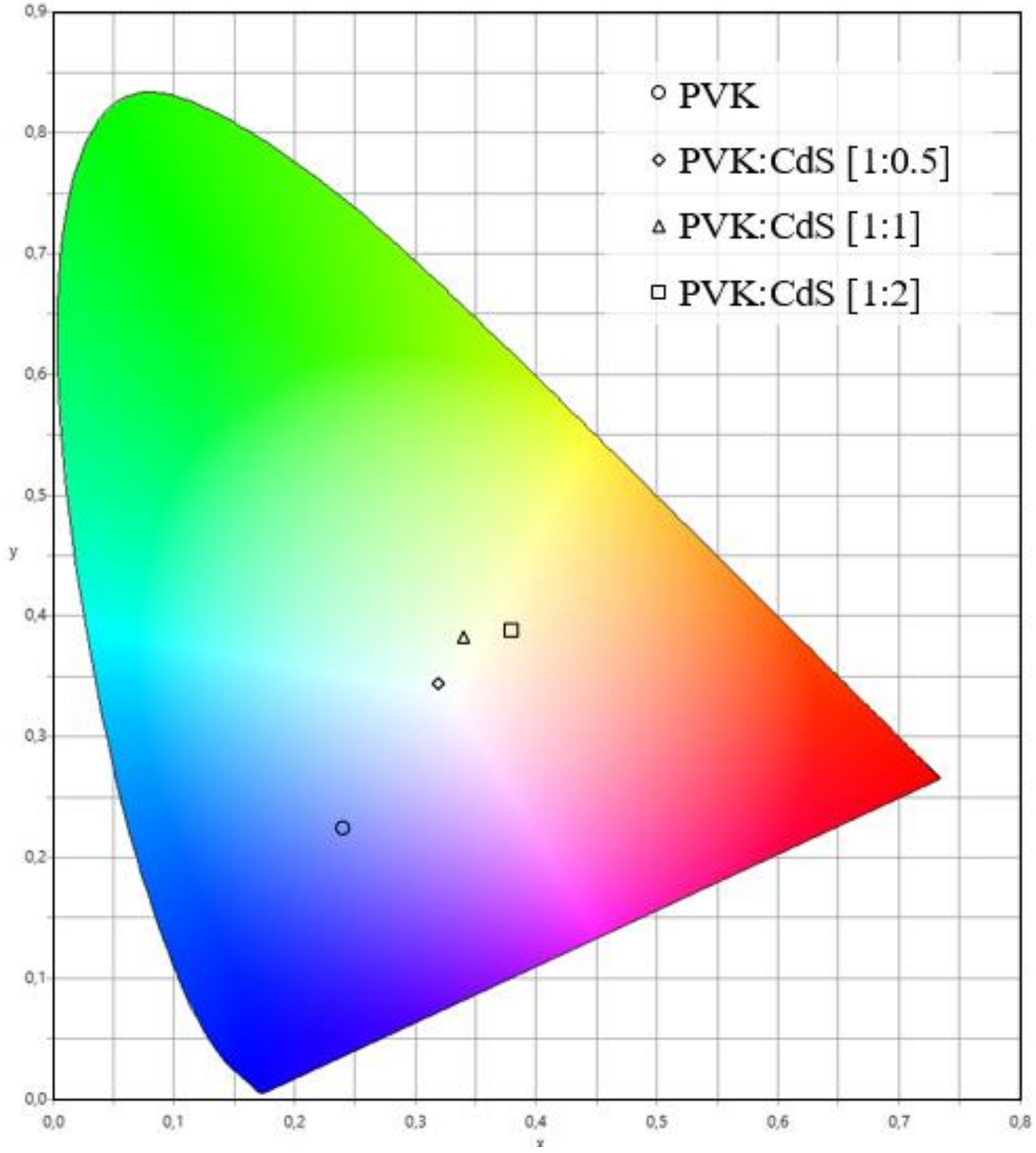

Figure S2. CIE coordinates of the hybrid LEDs with increasing CdS content: PVK circle, PVK:CdS [1:0.5] diamond, PVK:CdS [1:1] triangle and PVK:CdS [1:2] square.
